# Genomic Classification to Predict Survival in Metastatic Prostate Cancer: Development of Somatic Tumor Risk Assessment for Overall Survival-Prostate

**DOI:** 10.1200/PO-26-00021

**Published:** 2026-07-15

**Authors:** Martin W. Schoen, Jiannong Li, Sihang Zeng, Heena Desai, Ryan Hausler, Candace L. Haroldsen, Lukas Owens, Luca F. Valle, Ruth B. Etzoni, Timothy R. Rebbeck, Brent S. Rose, Michael J. Kelley, R. Bruce Montgomery, Nicholas G. Nickols, Matthew B. Rettig, Kosj Yamoah, Kara N. Maxwell, Isla P. Garraway

**Affiliations:** ^1^ Saint Louis University School of Medicine, St Louis, MO; ^2^ VA St Louis Healthcare System, St Louis, MO; ^3^ Department of Biostatistics and Bioinformatics, H. Lee Moffitt Cancer Center, Tampa, FL; ^4^ James A. Haley Veterans' Hospital, Tampa, FL; ^5^ Department of Biomedical Informatics and Medical Education, University of Washington, Seattle, WA; ^6^ Fred Hutchison Cancer Institute, Seattle, WA; ^7^ Puget Sound VA Healthcare System, Seattle, WA; ^8^ Medical Oncology Service, Corporal Michael Crescenz VA Medical Center, Philadelphia, PA; ^9^ Division of Hematology-Oncology, Department of Medicine, Perelman School of Medicine, University of Pennsylvania, Philadelphia, PA; ^10^ University of Utah, Salt Lake City, UT; ^11^ Veterans Affairs Salt Lake City Healthcare System, Salt Lake City, UT; ^12^ Department of Radiation Oncology, David Geffen School of Medicine at the University of California Los Angeles, Los Angeles, CA; ^13^ UCLA Jonsson Comprehensive Cancer Center, Los Angeles, CA; ^14^ Veterans Affairs Greater Los Angeles Healthcare System, Los Angeles, CA; ^15^ Department of Epidemiology, Harvard T. H. Chan School of Public Health, Boston, MA; ^16^ Division of Medical Oncology, Dana-Farber Cancer Institute, Boston, MA; ^17^ Veterans Affairs Boston Healthcare System, Boston, MA; ^18^ Veterans Affairs San Diego Healthcare System, San Diego, CA; ^19^ Department of Radiation Oncology, University of California, San Diego, San Diego, CA; ^20^ Department of Veteran Affairs, National Oncology Program, Washington, DC; ^21^ Durham VA Medical Center, Durham, NC; ^22^ Department of Medicine and Duke Cancer Institute, Duke University, Durham, NC; ^23^ Department of Hematology/Oncology, University of Washington, Seattle, WA; ^24^ Department of Hematology and Oncology, David Geffen School of Medicine at the University of California Los Angeles, Los Angeles, CA; ^25^ Department of Genetics, Perelman School of Medicine, Philadelphia, PA; ^26^ Department of Urology, David Geffen School of Medicine at the University of California Los Angeles, Los Angeles, CA

## Abstract

**PURPOSE:**

Tumor comprehensive genomic profiling (CGP) has revolutionized cancer care and identifies patients for biomarker-specific therapy. In metastatic hormone-sensitive prostate cancer (mHSPC), although individual genes are prognostic, no comprehensive genomic classification exists using CGP that accounts for combinations of alterations to inform prognosis. We developed a DNA-based CGP classification that is prognostic for overall survival (OS) and could inform treatment.

**METHODS:**

This was a retrospective cross-sectional study using multivariable models to develop a clinicogenomic prognostic risk classification in US veterans with synchronous mHSPC. The primary outcome was OS from time of metastasis.

**RESULTS:**

A total of 7,201 veterans with metastatic prostate cancer and CGP were identified. There were 2,484 veterans (median [IQR] age, 72 [67-77] years) with synchronous mHSPC and tissue CGP, which were divided into training and testing data sets. Sixteen genes associated with survival were identified, and favorable, intermediate, and unfavorable genomic prognostication groups were created based on the mortality risk to generate the Somatic Tumor Risk Assessment for OS-Prostate (STRATOS-P) classification. In a multivariable model, classification into intermediate and unfavorable groups was associated with increased mortality relative to the favorable group (adjusted hazard ratio [aHR], 1.54 [95% CI, 1.33 to 1.78]; aHR, 2.37 [95% CI, 1.97 to 2.485], respectively), demonstrating an average AUC of 0.83. In an external, nonveterans validation cohort, intermediate and unfavorable classifications were associated with increased mortality (aHR, 2.45 [95% CI, 1.87 to 3.21]; aHR, 4.37 [95% CI, 3.06 to 6.22], respectively) with an AUC of 0.79. The intermediate and unfavorable genomic prognostication groups were also associated with increased mortality across multiple disease states including synchronous and metachronous diagnoses, castration resistance, and analyte type.

**CONCLUSION:**

In metastatic prostate cancer, tumor DNA genomic alterations are prognostic for OS. The STRATOS-P classification is a validated prognostic tool that has the potential to guide decision making in mHSPC.

## BACKGROUND

Tumor DNA alterations are fundamental to understanding cancer biology. Disruption of tumor suppressor genes, such as *TP53*, carries prognostic importance^1,2^ while alterations in homologous recombination repair deficiency genes predict response to specific therapies, such as PARP inhibitors.^3^ In breast cancer, RNA-based gene expression assays, such as the 21-gene recurrence score (OncoType DX), are prognostic for the risk of recurrence^4^ and overall survival (OS).^5^ These tests inform clinical management, including the use of chemotherapy in early breast cancer.^6^ Similar integrative approaches in prostate cancer using RNA expression combined with clinical nomograms can improve prognostication and are being tested as potential predictive biomarkers.^7,8^

**CONTEXT**

**Key Objective**
Can comprehensive genomic profiling (CGP) be used to create a prognostic classification in metastatic prostate cancer?
**Knowledge Generated**
Using tumor sequencing from over 7,200 US veterans, a DNA-based genomic classification that stratifies patients with metastatic hormone-sensitive prostate cancer into favorable, intermediate, and unfavorable risk groups was created. This system, called Somatic Tumor Risk Assessment for Overall Survival-Prostate, prognosticates overall survival in multivariable models and was externally validated with strong prognostic performance.
**Relevance**
Classification of genomic alterations using existing CGP from routine clinical practice informs survival and may assist selection of therapy in metastatic prostate cancer.

Prostate cancer is the most common noncutaneous cancer in men with increasing incidence.^9,10^ Although metastatic disease is frequently lethal, survival has improved over time,^11^ and many men do not die from their disease.^12^ Given the heterogeneity in survival and increasing treatment options, stratifying patients into higher and lower risk groups could help to tailor treatment. In localized prostate cancer, where there are clinicopathologic risk-stratification systems,^13,14^ gene expression–based assays have been developed to classify the risk of metastatic progression,^7,8^ and multimodal AI models to predict benefit of androgen-deprivation therapy.^15,16^ In metastatic disease, risk stratification relies primarily on disease volume and timing of the onset of metastatic disease (synchronous *v* metachronous) to predict benefits of therapy.^17-20^ Recently, a 22-gene RNA expression score was found to be prognostic in metastatic disease and predicted benefit from docetaxel in *PTEN*-inactivated tumors.^21^ There are many genomic alterations in prostate cancer that inform prognosis and may be actionable.^22-26^

A wealth of data is collected in modern cancer care that includes patient characteristics, disease features, imaging, and comprehensive genomic profiling (CGP), many of which have not been integrated or used to their full clinical potential. CGP is recommended by ASCO, the Veterans Health Administration (VHA), and other national guidelines for all patients with metastatic prostate cancer.^27,28^ It is common for CGP to include analysis of over 300 genes, markers of microsatellite instability, and immunohistochemical tests. These data provide significant information about disease biology and may contribute to prognostication and/or prediction of response to therapies with little or no additional cost or burden on patients or clinicians.

The VHA serves over 16,000 veterans with metastatic prostate cancer, including more than 2,000 diagnosed with metastatic disease per year.^29^ As a part of the National Precision Oncology Program (NPOP), somatic DNA CGP has been made available for every veteran with metastatic prostate cancer at no cost to the veteran.^30,31^ With the comprehensive clinical data, a unique opportunity to classify tumor CGP into prognostic categories exists without the need for additional sample acquisition. Using CGP from 7,201 veterans with metastatic prostate cancer, we developed and validated a prognostic risk classification for patients with synchronous metastatic hormone-sensitive prostate cancer (mHSPC) and tested its performance in an independent cohort and multiple metastatic prostate cancer disease states and analytes.

## METHODS

### VA Data and Patient Population

The VHA Informatics and Computing Infrastructure was used to access the Corporate Data Warehouse and clinicopathologic data from the Veterans Affairs-Multi-OMICs Analysis Platform for Prostate Cancer (VA-MAPP) repository. Somatic tissue CGP with a commercially available platform (FoundationOne CDx or FoundationOne Liquid CDx; Foundation Medicine) was analyzed within the RESOLVE (Rate Elements Skewing Outcomes Linked to Veteran Equity) study via a data use agreement from NPOP. The study was deemed institutional review board (IRB) exempt by the VA Central IRB and analyses were performed in accordance with the Declaration of Helsinki. A waiver of consent was approved and results are reported according to Strengthening the Reporting of Observational Studies in Epidemiology guidelines.

**FIG 1.**
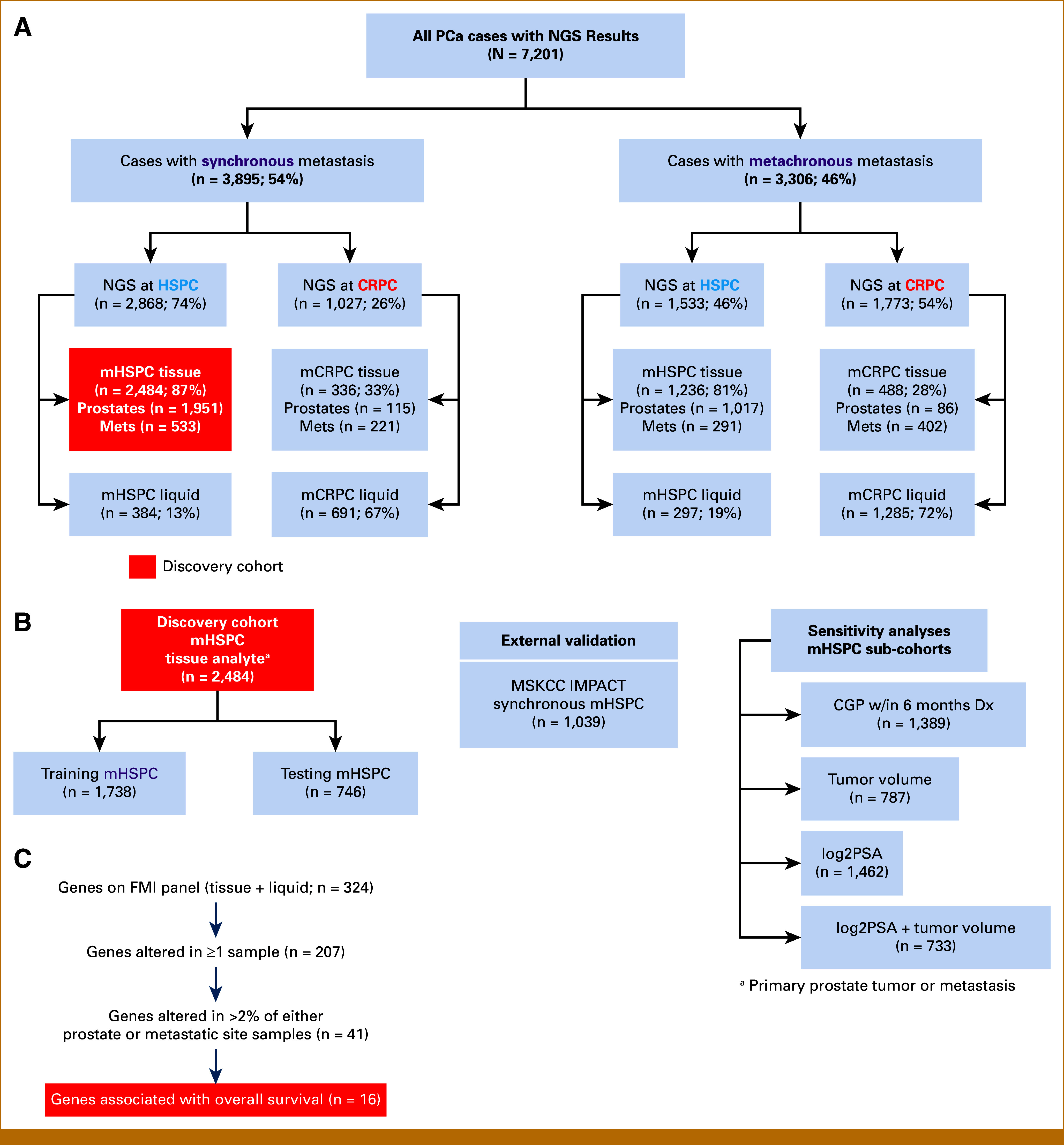
Cohort creation for genomic classification. (A) Complete categorization of all samples of patients with metastatic prostate cancer from NPOP. The date of sample acquisition was used to determine the state of disease. Patients with no evidence of prostate cancer before 12 months before metastatic diagnosis were considered to be synchronous while patients with a diagnosis of prostate cancer before 12 months were considered to be metachronous. Samples acquired from patients within 90 days before castration resistance were considered castration resistant while samples acquired more than 90 days before castration resistance were considered hormone sensitive. Tissue samples that were determined to be synchronous mHSPC were used to create the discovery cohort to initially classify genomic alterations. (B) Creation of the training and testing subgroups of patients with mHSPC in the discovery cohort and the primary validation cohorts of MSK-IMPACT and sensitivity analyses in specific cohorts of patients with synchronous mHSPC. (C) Selection of genes from Foundation Medicine Panel for inclusion in analysis. CGP, comprehensive genomic profiling; CRPC, castrate resistant prostate cancer; FMI, Foundation Medicine; mCRPC, metastatic castrate resistant prostate cancer; mHSPC, metastatic hormone-sensitive prostate cancer; MSK-IMPACT, Memorial-Sloan Kettering-Integrated Mutation Profiling of Actionable Cancer Targets; NGS, next generation sequencing; NPOP, National Precision Oncology Program; PCa, prostate cancer; PSA, prostate-specific antigen.

Veterans diagnosed with metastatic prostate cancer from 2003 to 2024 were identified by a natural language processing (NLP) algorithm^29^ and determined to be synchronous (diagnosis of prostate cancer <12 months before metastasis) or metachronous (diagnosis of prostate cancer ≥12 months before metastasis; Fig 1, Data Supplement, Table S1). At the time of sample collection, tumors were determined to be hormone sensitive if there was no evidence of castration resistant prostate cancer by a NLP algorithm before or up to 3 months after tumor sampling, otherwise tumors were castration resistant^32^ (Figure 1A, Data Supplement, Table S1). Samples were divided into CGP performed on tissue (prostate or metastasis) or plasma (liquid biopsy), yielding eight cohorts (Fig 1A, Data Supplement, Table S1). The date of diagnosis of metastatic disease was the start of observation (index date). Categorical variables, including age and gene alterations, that were selected for inclusion were summarized using frequencies and proportions. The primary outcome for classification was OS, determined from diagnosis of metastasis to death or censoring, which was October 4, 2024.

### MSK-IMPACT Validation Data

Patients with metastatic prostate cancer with tumor sequencing data derived from the Memorial-Sloan Kettering-Integrated Mutation Profiling of Actionable Cancer Targets (MSK-IMPACT)^33^ panel was obtained.^34^ In MSK-IMPACT, synchronous metastatic disease was identified if date of surgery (biopsy) was 1 year or less before metastases. Patients with only unspecified or male genital metastases were excluded. High-volume disease was determined if metastases included lung, pleura, liver, biliary tract, and CNS metastases. Time from metastasis to death was the primary outcome, and patient characteristics were determined across MSK-IMPACT data sets.^35^

### Creation of Genomic Prognostication Groups

The discovery cohort included veterans with synchronous mHSPC (n = 2,484) and was randomly split into training (n = 1,738) and test (n = 746) sets at a 2:1 ratio, with OS divided between the two groups (Fig 1B). Oncogenic somatic tumor DNA alterations including short variants, copy number alterations, and rearrangement variant calls were identified from CGP,^36^ and genes were selected if found at >2% frequency in either prostate or metastatic tissue. Genes selected for inclusion were significantly associated with OS with Benjamini-Hochberg^37^ adjusted *P* values of <.05 in the training set using a multivariable Cox proportional hazards model including age, prostate-specific antigen (PSA), and Charlson Comorbidity Index (CCI) as only these covariates were associated with OS.^38^

Unsupervised hierarchical clustering was performed on alterations associated with OS in the training set using Jaccard distance and the Ward.D2 linkage. Based on clustering and clinical relevance determined by expert review, genomic alterations were categorized into three groups using hazard ratios (HRs) of less than <1, >1-2, or >2. Veterans with multiple alterations were categorized into the highest-risk group. Additional prediction methods were performed using clinical variables with the following: (1) No inclusion of genomic data (2), using all genes in the FoundationOne panel, and (3) only the Somatic Tumor Risk Assessment for OS-Prostate (STRATOS-P) genomic classification. Each data set was evaluated using the following methods: (1) an elastic net Cox model, (2) random survival forest, and (3) gradient boosting to assess improved concordance.

### Statistical Analyses

The Kaplan-Meier method was used to estimate survival in the synchronous mHSPC discovery cohort and 1,389 veterans with synchronous mHSPC whose CGP was ordered within 6 months of metastatic diagnosis. Model performance for predicting OS from 1 to 5 years was evaluated by the mean time-dependent area AUC (tAUC) using the discovery cohort test set including age, PSA, CCI, year of diagnosis, volume of disease, and genomic classification. Missing values were treated as unknown.^39^ Model performance using MSK-IMPACT external data set was determined with mean tAUC from 1 to 5 years using age, volume of disease, and genomic classification. Model performance for predicting OS from 1 to 5 years was also evaluated by tAUC in seven other VA cohorts based on diagnosis, hormonal sensitivity, and analyte status (Fig 1B). A sensitivity analysis of patients with complete data available was conducted in the synchronous mHSPC discovery cohort including tumor volume from existing analyses^40^ and PSA. Statistical analyses were conducted using R software version 4.2.0^41^ and Python v3.10 using the scikit-survival package.^42^

## RESULTS

Demographics, comorbidities, and clinicopathologic features of the synchronous mHSPC discovery cohort (n = 2,484) are reported in Table 1. The median age (IQR) at diagnosis of synchronous mHSPC was 72 (66-77) years with 765 veterans (31%) self-identified as Black. The median (IQR) CCI was 2 (0-4) and BMI was 28.0 (24.6-31.6). The median (IQR) PSA at diagnosis was 47.6 ng/dL (14.9-176) and year of diagnosis was 2021 (IQR, 2019-2022). Tissue for analysis was obtained from the prostate in 1951/2,484 (78.5%) and from a metastatic site in 533/2,484 (21.5%; Fig 1). Sequencing was performed within 6 months of metastatic diagnosis in 1,389 (55.9%) of veterans at a median of 3.5 months (IQR, 0.5-22.2). The median OS was 59.4 months (95% CI, 54.3 to 64.4) and 39.0 months (95% CI, 34.4 to 43.5) in veterans sequenced within 6 months of metastasis.

**TABLE 1.** Baseline Characteristics of the Discovery Cohort of Patients With Synchronous Metastatic Hormone-Sensitive Prostate Cancer in All Patients, the Training Subgroup, and the Testing Subgroup

Clinical Characteristic	Overall (n = 2,484), No. (%)	Training (n = 1,738), No. (%)	Testing (n = 746), No. (%)	*P*
Age mPCa diagnosis				.9
<65	509 (20)	361 (21)	148 (20)	
65-74	1,040 (42)	727 (42)	313 (42)	
≥75	935 (38)	650 (37)	285 (38)	
Ethnicity				.2
Non-Hispanic	2,253 (91)	1,565 (90)	688 (92)	
Hispanic	142 (5.7)	106 (6.1)	36 (4.8)	
Other/unknown	89 (3.6)	67 (3.9)	22 (2.9)	
Race				.2
White	1,528 (62)	1,052 (61)	476 (64)	
Black	765 (31)	545 (31)	220 (29)	
Other/unknown	191 (7.7)	141 (8.1)	50 (6.7)	
CCI at PCa diagnosis				>.9
0-1	1,122 (45)	788 (45)	334 (45)	
2-3	609 (25)	422 (24)	187 (25)	
4+	753 (30)	528 (30)	225 (30)	
ADI year PCa diagnosis				.2
Median (IQR)	6 (3-8)	6 (3-8)	6 (3-8)	
Unknown	40	24	16	
BMI at mPCa diagnosis				>.9
Median (IQR)	28.0 (24.6-31.6)	28.0 (24.6-31.8)	27.9 (24.6-31.5)	
Unknown	606	415	191	
Military exposures				.15
Reported	494 (20)	332 (19)	162 (22)	
Marital status				>.9
Married	1,205 (49)	838 (48)	367 (49)	
Never married	335 (13)	236 (14)	99 (13)	
Other/unknown	944 (38)	664 (38)	280 (38)	
Tobacco use				.5
Reported	1,206 (49)	835 (48)	371 (50)	
PSA at mPCa diagnosis				.7
≤20	651 (26)	448 (26)	203 (27)	
20-100	729 (29)	520 (30)	209 (28)	
>100	704 (28)	494 (28)	210 (28)	
Unknown	400 (16)	276 (16)	124 (17)	
Gleason grade				.7
Grade 1	29 (1.5)	20 (1.5)	9 (1.6)	
Grade 2	104 (5.5)	69 (5.1)	35 (6.2)	
Grade 3	183 (9.6)	123 (9.2)	60 (11)	
Grade 4	499 (26)	353 (26)	146 (26)	
Grade 5	1,091 (57)	776 (58)	315 (56)	
Unknown	578	397	181	
Histology				.7
Carcinoma/adeno	2,413 (97)	1,690 (97)	723 (97)	
Neuroendocrine/small cell	38 (1.5)	27 (1.6)	11 (1.5)	
Other/unknown	33 (1.3)	21 (1.2)	12 (1.6)	
Primary tumor treatments (reported)				
Radiation	818 (33)	570 (33)	248 (33)	.8
ADT	1,356 (55)	948 (55)	408 (55)	>.9
AA/ARPI	2,339 (94)	1,643 (95)	696 (93)	.3
Taxane	727 (29)	520 (30)	207 (28)	.3
Vital status				
Deceased	1,001 (40)	700 (40)	301 (40)	>.9
PCa-specific mortality	204 (75)	141 (76)	63 (72)	.5
OS, median (IQR)	28.8 (16.0-49.8)	28.4 (15.9-49.2)	29.6 (16.1-51.2)	.4

Abbreviations: AA, antiandrogen; ADI, area deprivation index, state block level group; ADT, androgen deprivation therapy; ARPI, androgen receptor inhibitor; CCI, Charlson Comorbidity Index; mPCa, metastatic prostate cancer; PCa, prostate cancer; PSA, prostate-specific antigen.

**FIG 2.**
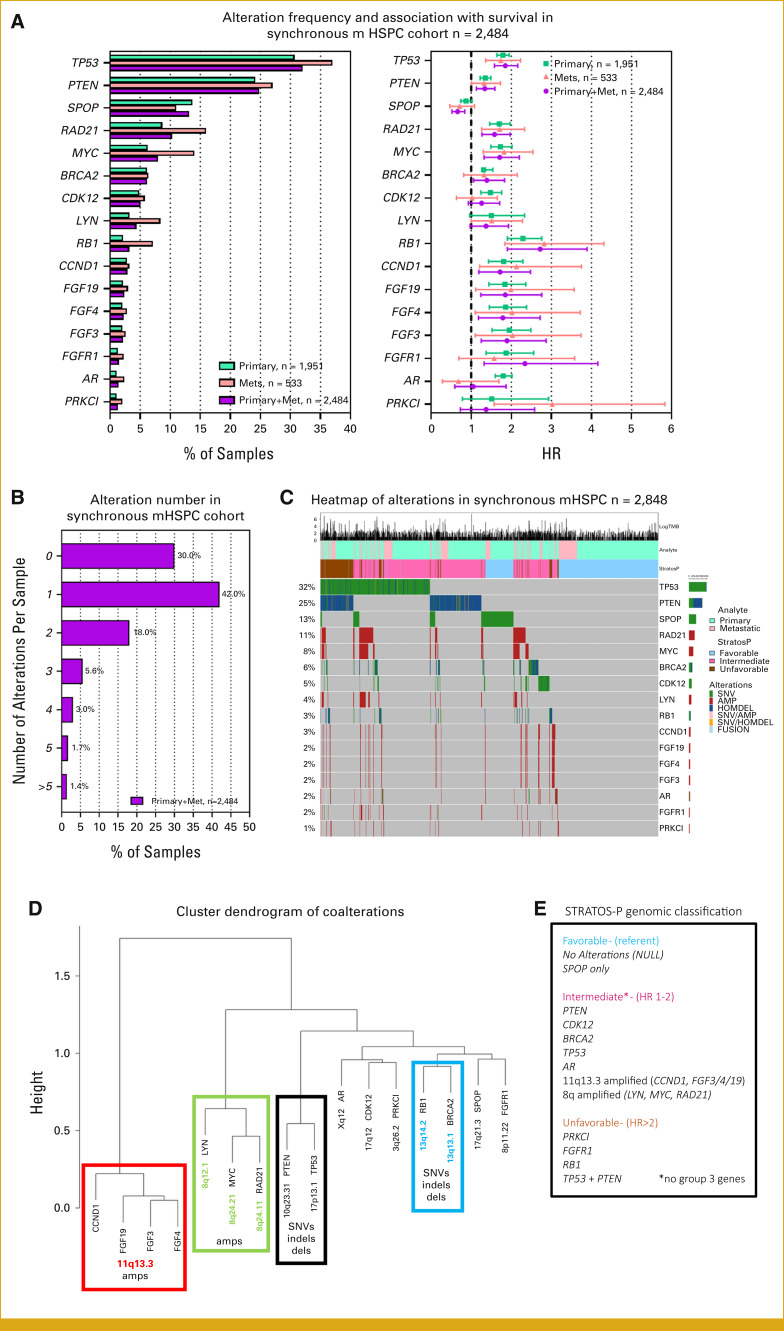
The incidence and types of alterations in patients in synchronous metastatic hormone-sensitive prostate cancer. (A) Incidence of genetic alterations based on site of biopsy in 16 genes selected and hazard ratio for OS in multivariable model including age and Charlson Comorbidity Index. (B) Distribution of the number of 16 gene alterations patients with synchronous metastatic hormone-sensitive prostate cancer. (C) Heatmap of alterations by gene and type synchronous metastatic hormone-sensitive prostate cancer. (D) Cluster dendrogram of alterations to display alterations that occur frequently together. (E) Final grouping of DNA alterations in prognostic categories. HR, hazard ratio; mHSPC, metastatic hormone-sensitive prostate cancer; OS, overall survival; SNV, single nucleotide variant.

The results of CGP were used in the discovery cohort to identify pathogenic alterations prognostic for OS (Fig 1C). The association of alterations with OS was tested in a multivariable model and compared with samples with no alteration in that gene. After applying exclusion criteria, 16 genes were identified (Figs 1C and 2A, Data Supplement, Tables S2A and S2B). These genes include the general tumor suppressor genes *TP53*, *PTEN*, and *RB1*, and genes involved in cell cycle regulation (*CCND1*, *CDK12*), DNA repair (*BRCA2*, *RAD21*), cell growth (*MYC*, *FGFR1*, *FGF3*, *FGF4*, *FGF19*, *PRKC1*, *LYN*), and androgen signaling (*AR*, *SPOP*). In the synchronous mHSPC discovery cohort, 70.1% (1,741) of tumors sequenced had at least one oncogenic alteration, 28% (694) had two or more alterations, and 11.6% (289) had three or more (Fig 2B); 30% had no alteration in any of the 16 genes.

To classify genes, analysis of coalterations and mutual exclusivity was performed (Figs 2C and 2D, Data Supplement, Table S2C). The most common coalteration was *TP53* and *PTEN*, likely related to disease biology, as these genes are on different chromosomes (17p13.1 and 10q23.31, respectively). In contrast, other coalterations likely occur because of common coamplifications in prostate cancer, namely amplifications of 8q that involve *RAD21*, *LYN*, and *MYC* and 11q13.3, resulting in alterations of *CCND1, FGF19, FGF3*, and *FGF4*. Unsupervised hierarchical clustering of samples based on alterations confirmed these groupings (Fig 2D).

**FIG 3.**
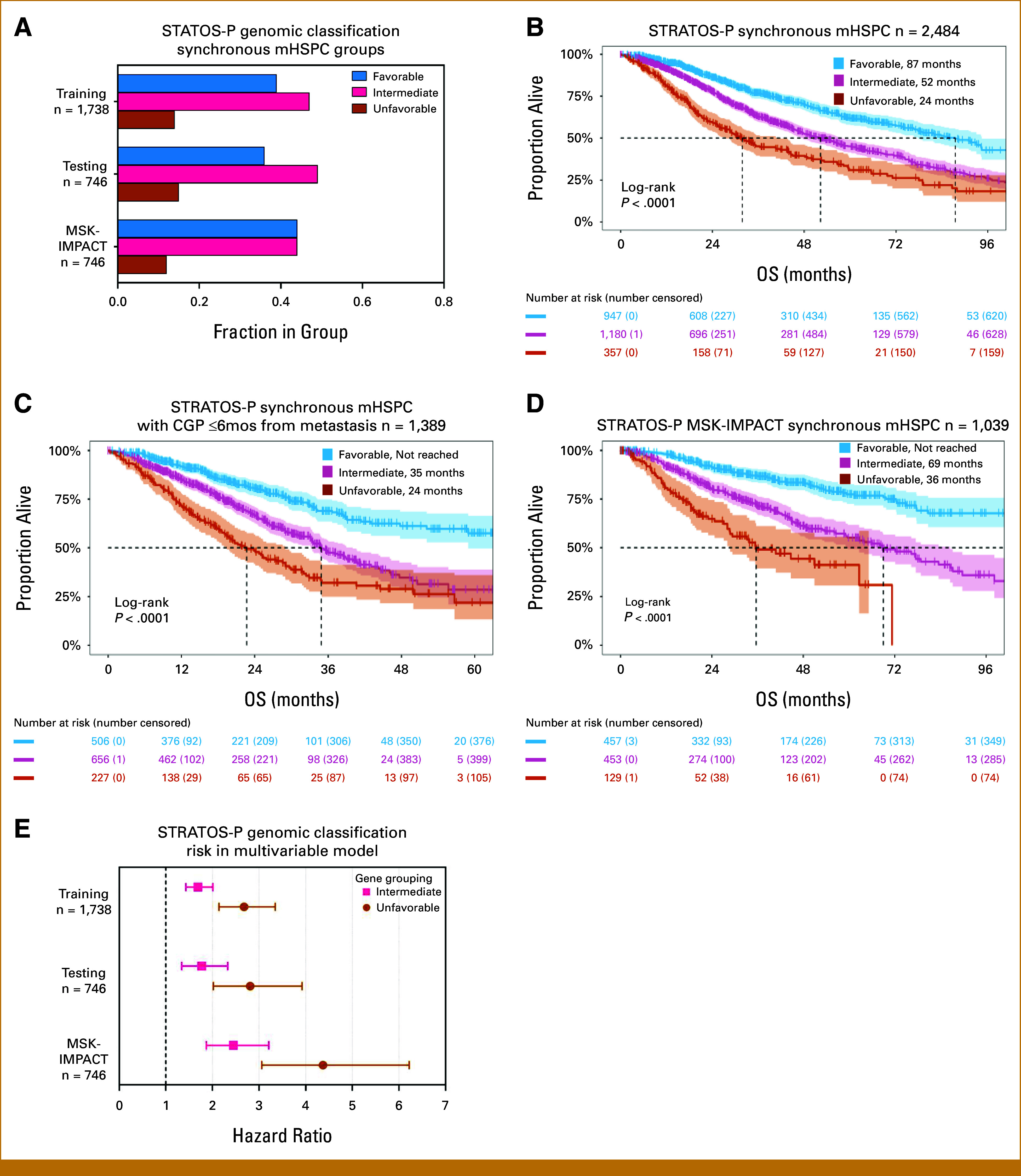
Distribution, OS, and risk of mortality based on genomic classification. (A) Frequency of genomic risk classification in synchronous mHSPC in Veterans and Memorial Sloan Kettering-IMPACT. (B) OS in all patients with synchronous mHSPC in the VHA based on STRATOS-P genomic classification. (C) OS in all patients with synchronous mHSPC who have genomic sequencing ordered within 6 months of diagnosis in the VHA based on the STRATOS-P genomic classification. (D) OS in patients identified with synchronous mHSPC in MSK IMPACT based on STRATOS-P genomic classification. (E) Adjusted hazard ratio for death in synchronous mHSPC training, testing cohorts, and MSK-IMPACT based on STRATOS-P genomic classification in the multivariable model. CGP, comprehensive genomic profiling; mHSPC, metastatic hormone-sensitive prostate cancer; MSK-IMPACT, Memorial Sloan Kettering-Integrated Mutation Profiling of Actionable Cancer Targets; OS, overall survival; STRATOS-P, Somatic Tumor Risk Assessment for OS-Prostate; VHA, Veterans Health Administration.

Clustering, genomic colocalization, and HRs from the multivariable model were used to group genes (Fig 2E). Genes with a HR of <1 or no alteration were grouped in the favorable risk category. One or more of the genes with HR of 1-2 and the presence of coalterations were included in the intermediate-risk group. The unfavorable-risk group included genes with HR > 2 and the frequent coalteration of *TP53* and *PTEN*, which was present in 9.5% (234/2,484). Veterans were classified by the most adverse alteration present. In the synchronous mHSPC discovery cohort, 947/2,484 (38%) of veterans were favorable risk while 1,180/2,484 (48%) and 357/2,484 (14%) were classified as intermediate and unfavorable risk, respectively (Fig 3A). Additional methods of classification showed no improvements in AUC; therefore, the Cox model–based selection was used (Data Supplement, Table S3). Unadjusted OS of the discovery cohort was significantly lower in the intermediate (median OS, 52 months, *P* < .001) and unfavorable (median, OS 31 months, *P* < .001) groups compared with the favorable group (median OS, 87 months; Fig 3B). In veterans with sequencing <6 months after diagnosis, unadjusted OS remained significantly lower in the intermediate (median OS, 36 months, *P* < .001) and unfavorable (median OS, 24 months, *P* < .001) groups compared with the favorable group (median OS, not reached; Fig 3C).

The genomic classification was evaluated in the training data set of 1,738 synchronous mHSPC veterans to determine its performance in the multivariable model with age and CCI. In these veterans, the adjusted HR (aHR) for mortality was 1.55 (95% CI, 1.30 to 1.84, *P* < .001) and 2.30 (95% CI, 1.83 to 2.88, *P* < .001) in the intermediate and unfavorable groups, respectively. In the test set of 746 veterans held out from the discovery cohort of synchronous mHSPC, the aHR for mortality was 1.59 (95% CI, 1.20 to 2.11) and 2.73 (95% CI, 1.95 to 3.82) in the intermediate and unfavorable groups (Fig 3C, Table 2), respectively. The average tAUC was 0.83 using the full Cox model.

**TABLE 2.** Multivariable Cox Model Features for Patients With Synchronous Metastatic Hormone-Sensitive Prostate Cancer Based on Genomic Classification, Age, CCI, and Tumor Volume That Was Available

Variable	Training, n = 1,738		Testing, n = 746		MSK IMPACT, n = 1,039	
	No. (%)	HR (95% CI)	No. (%)	HR (95% CI)	No. (%)	HR (95% CI)
Age <65	361 (21)	Ref	148 (20)	Ref	492 (47)	Ref
Age 65-74	727 (42)	1.18 (0.96 to 1.45)	313 (42)	1.46 (1.04 to 2.06)	382 (37)	1.29 (0.99 to 1.68)
Age ≥75	650 (37)	1.40 (1.12 to 1.74)	285 (38)	1.63 (1.13 to 2.33)	165 (16)	2.61 (1.93 to 3.53)
CCI 0-1	788 (45)	Ref	334 (45)	Ref	NA	
CCI 2-3	422 (24)	1.35 (1.12 to 1.65)	187 (25)	1.02 (0.75 to 1.39)	NA	
CCI 4+	528 (30)	1.71 (1.43 to 2.03)	225 (30)	1.80 (1.37 to 2.36)	NA	
PSA <20	448	Ref	203	Ref	NA	
PSA 20-100	520	1.05 (0.86 to 1.29)	209	1.04 (0.76 to 1.42)	NA	
PSA>100	494	1.38 (1.13 to 1.68)	210	1.41 (1.04 to 1.92)		
Unknown	276	0.71 (0.50 to 0.99)	124	0.79 (0.47 to 1.34)	NA	
Low volume	401 (23)	Ref	177 (24)	Ref	782 (75.3)	Ref
High volume	605 (35)	1.53 (1.25 to 1.87)	270 (36)	2.31 (1.66 to 3.3)	257 (24.7)	3.78 (3.00 to 4.76)
Unknown	732 (42)	0.84 (0.68 to 1.05)	299 (40)	1.28 (0.89 to 1.83)	0 (0)	
Diagnosis year				1.27 (1.18 to 1.37)		
Favorable	681 (39)	Ref	266 (36)	Ref	457	Ref
Intermediate	815 (47)	1.65 (1.39 to 1.97)	365 (49)	1.68 (1.27 to 2.24)	453	2.19 (1.67 to 2.88)
Unfavorable	242 (14)	2.44 (1.95 to 3.07)	115 (15)	2.99 (2.12 to 4.20)	129	3.92 (3.00 to 4.75)

AUC	Testing, n = 746		MSK IMPACT, n = 1,014	
Time (months)	Age + CCI	Full Model^a^	Age	Full Model^b^
12	0.69	0.82	0.65	0.81
24	0.69	0.80	0.64	0.79
36	0.71	0.83	0.63	0.78
48	0.73	0.88	0.65	0.78
60	0.72	0.88	0.64	0.78
Average AUC	0.71	0.83	0.64	0.79

NOTE. Results are shown for the training subgroup, the testing subgroup, and with MSK-IMPACT. Time-dependent and average AUC measurements between the training subgroup and both the testing subgroup and MSK-IMPACT with volume of disease and genomic classification are shown.

Abbreviations: AUC, area under the receiver operating characteristic curve; CCI, Charlson Comorbidity Index; MSK-IMPACT, Memorial Sloan Kettering-Integrated Mutation Profiling of Actionable Cancer Targets; NA, not available; PSA, prostate-specific antigen; Ref, reference.

^a^ Included age, CCI, PSA, volume of disease, year of diagnosis, and genomic classification.

^b^ Included age, volume of disease, and genomic classification; 25 patients were not included in AUC calculation because of longer follow-up time than observed in the training set.

In the MSK-IMPACT validation cohort of 1,039 patients with synchronous mHSPC (Data Supplement, Table S4), the median survival was 89.8 months (95% CI, 72.1 to 107.4; Fig 3D). The favorable group included 44.0% (457/1,039) patients, the intermediate group included 43.6% (453/1,039), and the unfavorable group 12.4% (129/1,039; Fig 3A). Using age and genomic classification, the aHR for mortality in MSK-IMPACT of the intermediate group was 2.45 (95% CI, 1.87 to 3.21) and 4.37 (95% CI, 3.06 to 6.22) in the unfavorable group (Table 2, Fig 3E). The average tAUC over 12-60 months was 0.79 between the discovery cohort and MSK-IMPACT (Table 2).

In 1,389 veterans who had CGP within 6 months, mortality was slightly higher compared with the overall cohort with an aHR of 1.90 (95% CI, 1.52 to 2.38) and 3.16 (95% CI, 2.44 to 4.11) in the intermediate and unfavorable groups, respectively. The tAUC was 0.72 at 12 months (0.68-0.75) in the test set in those with sequencing within 6 months. Additional sensitivity analyses in veterans with known tumor volume (n = 787), PSA at diagnosis (n = 1,462), or both (n = 733). Adding tumor volume improved the tAUC at 12 months to 0.79, and adding PSA did not change the tAUC at 12 months, also 0.77 (Data Supplement, Table S5 and Fig S1).

**FIG 4.**
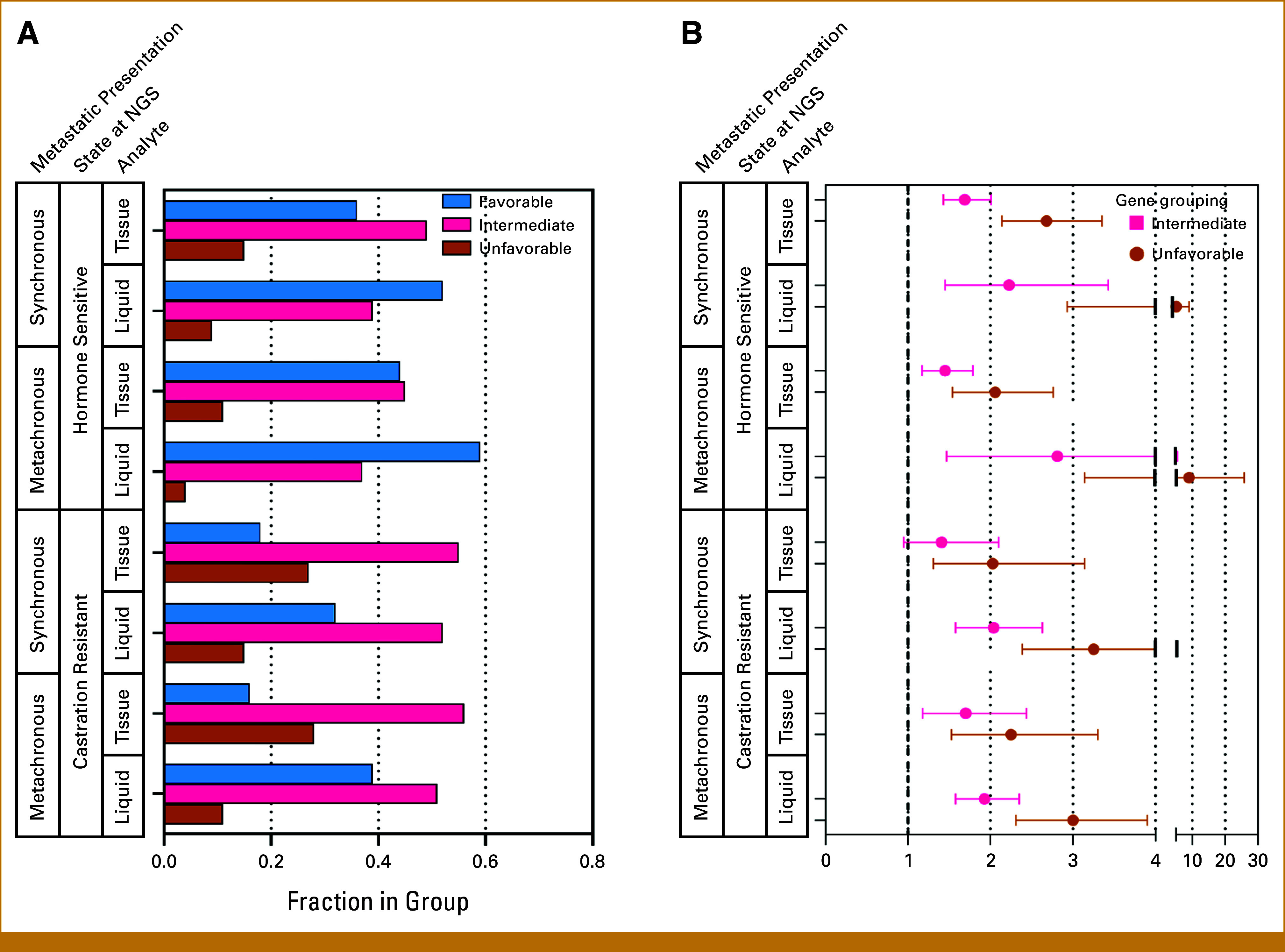
Analyses from VHA validation cohorts. (A) Incidence of alteration groups based on stage at biopsy, tissue versus liquid, and synchronous versus metachronous presentation. (B) Adjusted hazard ratio for validation cohorts based on disease state at the time of sequencing, tissue versus liquid biopsy, synchronous or metachronous, and type of biopsy. NGS, next generation sequencing; VHA, Veterans Health Administration.

The genomic classifier was assessed across other metastatic presentations, hormonal sensitivity, and sequencing analytes (Data Supplement, Table S1). In 384 veterans with synchronous mHSPC and a liquid biopsy, mortality was similar to the tissue analyte with an aHR of 2.23 (95% CI, 1.45 to 3.43) and 5.13 (95% CI, 2.93 to 8.97) in the intermediate and unfavorable groups, respectively, and the tAUC was 0.71 at 12 months (0.71-0.77; Fig 4, Data Supplement, Table S6). In veterans with synchronous metastases, CGP was obtained during castration resistance (tissue, n = 336; liquid biopsy, n = 691); the genomic classification remained associated with OS with acceptable performance in tissue (tAUC 0.70 [0.69-0.73]) and in liquid biopsy (tAUC 0.74 [0.70 to 0.78]) at 12 months. In veterans with a metachronous mHSPC sample, genomic classification was associated with mortality when either tissue (n = 1,236) or liquid (n = 297) was analyzed (tAUC 0.70 [0.63-0.77] and 0.79 [0.75-0.84], respectively). In veterans with a metachronous metastatic castrate resistant prostate cancer (mCRPC) sample, the genomic classification was associated with mortality when either tissue (n = 488) or liquid (n = 1,285) was analyzed (tAUC 0.70 [0.63-0.77] and 0.72 [0.68-0.78], respectively).

## DISCUSSION

This large, multicenter study demonstrates that a genomic classification of somatic DNA CGP provides powerful, reproducible prognostic information in metastatic prostate cancer. To date, CGP is not prognostic in synchronous mHSPC because there is no validated classification system. Our genomic classification, termed STRATOS-P, differentiates risk using a large commercially available panel. The use of existing CGP for prognosis without additional testing helps to understand heterogeneity in survival and has the potential to influence therapeutic decisions and tailor clinical trials for escalation/de-escalation strategies.

STRATOS-P was trained and validated in veterans with synchronous mHSPC and nonveterans with synchronous mHSPC from MSK-IMPACT. Sensitivity analyses restricting to synchronous mHSPC with CGP within 6 months of diagnosis to account for immortal time bias showed similar results. The performance was similar when applied to patients with metachronous mHSPC and mCRPC, demonstrating the robustness of STRATOS-P in different clinical scenarios. To date, no other clinical variable had as much ability to differentiate risk of mortality in various disease states, underscoring the importance of DNA genomic alterations in prognosis. It is important that STRATOS-P performed well in liquid biopsies because tumor tissue may be either unavailable or difficult to obtain.

Given the increase in metastatic prostate cancer incidence and treatment options, it is important to consider tumor heterogeneity.^9^ The combination of clinical and genomic features contribute to the promise of precision oncology to optimize treatment that could prolong survival, while considering quality of life and adverse events. As prostate cancer is a disease of aging, estimation of prognosis is especially important for frail patients or those at a risk of cardiac events to avoid overtreatment. In frail patients with low-risk metastatic prostate cancer, combination therapies may have little effect on mortality and may increase adverse events, as has been shown in trials of fit patients.^43^

Because of its retrospective design, this study is subject to unmeasured confounding. Veterans who received CGP may have different characteristics from other patients with mHSPC, biasing results. Furthermore, because CGP historically only affected treatment for mCRPC, many patients had CGP several months after metastatic diagnosis. This immortal time and left truncation biases were addressed through an analysis of patients sequenced within 6 months of diagnosis. Also, patients were identified as metastatic through NLP of VHA records and may include a population of patients with regional metastases.^29^ The clinical model used demonstrates that the prognostic importance of genomic alterations in mHSPC only includes patients with synchronous disease and does not include features such as Gleason score and visceral metastases. However, we did perform a sensitivity analysis using tumor volume showing improved performance. Incorporation of these variables may further improve the prognostic accuracy of clinical models of mHSPC. It is important to note that many patients may not have tissue available or that the CGP may fail, limiting the utility to prognosticate based on genomics. Additionally, other methods of genomic assessment, such as percent genome altered, are not clinically available on the sequencing panel used in this study but could add additional prognostic information.^44,45^ Finally, veterans may have received treatment outside of the VHA, creating uncertainty in the timing/accuracy of diagnosis and comorbid conditions.

The study has several strengths, most notably its large sample size. The ability to perform discovery and validation of genomic alterations in over 7,000 patients is important for creating a comprehensive classification. The large cohort allows for management of coalterations and an understanding of the interactions that commonly occur in tumors. The validation of the classification in other VA data and the external MSK-IMPACT data set increases the reliability of our findings. The use of existing CGP results without additional sampling or cost will also increase the ability to apply these findings in practice and clinical trials.

In conclusion, the STATOS-P genomic classification using DNA alterations from CGP is prognostic and was validated in internal VHA data and the external MSK-IMPACT cohort. This classification does not require additional tissue procurement or sequencing because it uses data obtained as part of contemporary clinical practice. In combination with clinical variables, STRATOS-P categorizes patients with prostate cancer into distinct prognostic risk groups. These groups could be used to inform shared decision making around treatment options and for prospective studies to evaluate disease risk combined with clinical variables to optimize treatment.

## Data Availability

A data sharing statement provided by the authors is available with this article at DOI https://doi.org/10.1200/PO-26-00021.
